# Comparison of outcomes of unilateral recession-resection as primary surgery and reoperation for intermittent Exotropia

**DOI:** 10.1186/s12886-017-0512-5

**Published:** 2017-07-05

**Authors:** Young Bok Lee, Dong Gyu Choi

**Affiliations:** Department of Ophthalmology, Hallym University College of Medicine, Kangnam Sacred Heart Hospital, 948-1 Daerim1-dong, Youngdeungpo-gu, Seoul, 150-950 Korea

**Keywords:** Unilateral recession-resection, Dose-effect ratio, Intermittent exotropia, Surgical success rate

## Abstract

**Background:**

The aim of this study was to compare the primary surgery and reoperation outcomes of unilateral lateral rectus recession and medial rectus resection (R&R) for intermittent exotropia.

**Methods:**

We retrospectively reviewed the medical records of 80 patients, all of whom had undergone unilateral R&R for intermittent exotropia as a primary surgery or reoperation and been followed-up on postoperatively for 6 months or more. The patients were divided into two groups: unilateral R&R as primary surgery (group A, 44 patients) and unilateral R&R as reoperation (group B, 36 patients). The outcome measures were postoperative angle of deviation, surgical success rate, and mean dose-effect ratio (PD/mm, corrected angle of deviation / sum of amount of recession of lateral rectus and of resection of medial rectus). Surgical success was defined as exo- or esodeviation within 8 PD.

**Results:**

The mean postoperative follow-up duration was 49.91 ± 14.83 months in group A and 43.17 ± 26.91 months in group B (*p* = 0.160). The mean angles of deviation at postoperative 1 day were −5.18 PD (overcorrection) in group A and −5.28 PD in group B (*p* = 0.932). However, there was a significant difference in the mean angle of deviation between the two groups at each visit from postoperative 3 months to final follow-up (*p* < 0.05): in short, group A had become more exotropic than group B. And the surgical success rate was higher in group B than in group A at each visit from postoperative 12 months to final follow-up (47.7% in group A and 83.3% in group B at final follow-up) (*p* < 0.05). The mean dose-effect ratio at 6 months after surgery was 1.89 ± 0.58 PD/mm in group A and 2.26 ± 0.32 PD/mm in group B (*p* = 0.001).

**Conclusions:**

Unilateral R&R as reoperation presented better results for the surgical treatment of recurrent exotropia, showing a smaller exodrift pattern and higher surgical success rates compared with R&R as a primary surgery. The mean effect per millimeter (the mean dose-effect ratio, PD/mm) of R&R as reoperation was significantly greater than that of R&R as primary surgery at postoperative 6 months. These results could serve as useful guidelines in the planning of surgical correction for primary and recurrent exotropia.

## Background

Intermittent exotropia is a common type of strabismus among East Asians [1–4]. The most commonly utilized surgical procedures for intermittent exotropia are bilateral lateral rectus (LR) recession and unilateral LR recession and medial rectus (MR) resection (R&R) [5]. Burian et al. proposed an exotropia classification based on differences of deviation at distance and near, and recommended different surgical methods for the different types [5–8]. They suggested that R&R is effective for exotropia of the basic or pseudo-divergence excess type and that bilateral LR recession is more suitable for the true divergence excess type [5–8].

Although intermittent exotropia can be improved and controlled by primary surgery in many instances, postoperative exodrift and recurrence are common, in which cases reoperation might be required [9–13]. The surgical intervention in cases of recurrent exotropia depends on the primary surgery [14]. Bilateral or unilateral MR resection might be performed on patients having previously undergone bilateral LR recession; or, patients on whom R&R was previously performed might undergo, as reoperation, LR recession or R&R on the contralateral eye. Some studies have shown better surgical results and lower recurrence rates for recurrent exotropia after reoperation [15, 16]. In another study, Kim and Kim reported that a lower degree of exodrift was observed after R&R as reoperation compared with R&R as primary surgery [17]. However, they did not compare the surgical success and dose-effect ratio of R&R between primary surgery and reoperation. Therefore, in the present study, we compared the surgical outcomes and dose-effect ratios (PD/mm) of unilateral R&R as primary surgery and reoperation for intermittent exotropia.

## Methods

A retrospective review of medical records was performed on the patients with intermittent exotropia who underwent unilateral R&R as a primary surgery or reoperation. The minimum follow-up period after surgery was 6 months. This study was reviewed and approved by the Institutional Review Board of the Hallym University Medical Center (2014–12-176).

All of the participating patients had been diagnosed with intermittent exotropia of the basic type according to Burian’s classification [6]. We assigned the patients to two groups: group A, those who had undergone unilateral R&R on the non-dominant eye as a primary surgery for exotropia, and group B, those who had undergone unilateral R&R as a reoperation for residual or recurrent exotropia.

The exclusion criteria were as follows: (1) a history of previous strabismus surgery by another surgeon, (2) A- or V-pattern exotropia, (3) R&R with concurrent horizontal muscle transposition for correction of vertical deviation, (4) trauma, (5) paralytic or restrictive exotropia, (6) other ocular diseases, or (7) systemic diseases such as Down syndrome or cerebral palsy.

### Preoperative examinations

The following preoperative characteristics were reviewed and analyzed: sex, age at surgery, best-corrected visual acuity, refractive error, mean angle of deviation at distance and near, stereopsis, lateral incomitance, amblyopia, presence of vertical deviation, dissociated vertical deviation (DVD), and oblique muscle dysfunction (Table 2). Lateral incomitance was defined as a change of 5 PD or more in right or left gaze from the primary position. Amblyopia was defined as a difference of 2 lines or more between monocular best-corrected visual acuities. Vertical deviation was defined as hypertropia or hypotropia with 5 PD or more at the primary position.

We performed complete ophthalmologic examinations before the surgery. We measured the visual acuity using a Snellen visual acuity chart. The decimal notation on recorded visual acuity was converted to a logarithm of the minimum angle of resolution (Log MAR) units using the conversion chart. Cycloplegic refraction was conducted using 1% cyclopentolate chloride. We conducted alternate prism cover test with an accommodative target at distance (6 m) and near (0.33 m) in all 9 positions of gaze. If the exodeviation at distance was larger than 10 PD compared with that at near, one eye was occluded for 1 h. The alternate prism cover test was repeatedly conducted. The degree of stereopsis was measured using Titmus Stereotest at near (0.3 m) (Stereo Optical Co., Inc., Chicago, IL, USA). We performed Worth-4-dot test at distance (6 m). The results were classified as follows: (1) fusion and (2) no fusion, composed of suppression and diplopia.

### Strabismus surgery

A single surgeon (Dong Gyu Choi) conducted all of the surgeries according to the formula modified from the surgical table proposed by Parks (Table 1) [18]. R&R was performed on the non-dominant eye in group A and on the unoperated eye in group B. In group B, all patients had undergone the unilateral R&R as primary surgery for intermittent exotropia.

**Table 1:** Surgical table of R&R as primary surgery or reoperation for intermittent exotropia

Distance deviation (PD)	LR recession (mm)	MR resection (mm)
20	5.0	4.0
25	6.0	5.0
30	7.0	5.5
35	7.5	6.0
40	8.0	6.5
45	8.5	6.5

*R&R* lateral rectus recession and medial rectus resection, *PD* prism diopters, *LR* lateral rectus muscle, *MR* medial rectus muscle

### Postoperative management

The angle of deviation was measured at postoperative 1 day, 1 week, 1, 3, 6 months, 1 and 2 years, and at final follow-up. The subjective diplopia was examined, and any abnormalities in duction and version were checked. If the patients had developed diplopia or esodeviation, full-time alternate patching was prescribed and continued until the diplopia or esodeviation was resolved. We defined the surgical success as ocular alignment within 8 PD. For the precise estimation of surgical success, any patient whose stereoacuity had 40 s of arc preoperatively and changed worse after surgery was regarded as a monofixator and excluded from the surgical success, in spite of the postoperative esodevation ≤8 PD. Overcorrection was defined as esodeviation over 8 PD, and undercorrection, as exodeviation over 8 PD.

### Outcome measures

The primary outcome measures included the surgical success rates based on the postoperative angle of deviation at distance and near as well as the sensory status determined by Titmus stereotest and Worth-4-dot test (W4D), which were compared between the two groups. The secondary outcome measure was the dose-effect ratio. For the purpose of measuring the dose-effect ratio of R&R, we defined the two terms: the dose was the sum of the amount of recession of lateral rectus (LR) and of resection of medial rectus (MR); the effect was the corrected angle of deviation, obtained by subtracting the postoperative 6 months angle of deviation from the preoperative angle of deviation. Then, we calculated the ratio of the effect to the dose (the dose-effect ratio).

### Statistical analysis

We performed the statistical analysis with SPSS software, version 12.0 K (SPSS Inc., Chicago, IL, USA). Using the Mann–Whitney *U* test and Pearson chi-square test, we compared the values between the two groups. The associations between the values were evaluated by Pearson’s correlation analysis. A *P*-value less than 0.05 was considered to be statistically significant.

## Results

Eighty (80) patients who had undergone R&R surgery for intermittent exotropia were enrolled in this retrospective study. Among them, 44 patients (group A) had undergone R&R on the non-dominant eye as a primary surgery, and 36 patients (group B) R&R as a reoperation. The patient characteristics were described in Table 2. The postoperative mean follow-up period was 49.91 ± 14.83 months (20–74 months) in group A and 43.17 ± 26.91 months (11–147 months) in group B (*p* = 0.160). The mean age at primary surgery in group A and at second surgery in group B was, respectively, 71.70 ± 30.59 months and 107.31 ± 40.53 months (*p* = 0.000). The preoperative angle of exodeviation at distance was 29.43 ± 6.75 PD and 25.28 ± 3.57 PD in groups A and B, respectively (*p* = 0.001). There was no difference between the groups in sex, refractive error, vertical deviation, DVD, amblyopia, or oblique muscle dysfunction (*p* > 0.05).

**Table 2:** Preoperative demographic data on groups A and B

	Group A (*n* = 44)	Group B (*n* = 36)	*P* value
Age at surgery (months)	71.70 ± 30.59	107.31 ± 40.53	0.000^*^
Sex (M/F)	23/21	17/19	0.653^†^
Preoperative angle of exodeviation (PD)			
Distance	29.43 ± 6.75	25.28 ± 3.57	0.001^*^
Near	29.43 ± 6.02	26.94 ± 3.64	0.026*
Stereopsis (seconds of arc)	301.82 ± 546.96	309.17 ± 467.81	0.949*
Good (≤ 100 s of arc, %)	63.6%	66.7%	0.777^†^
Fusion on W4D test at distance (n, %)	29 (65.9%)	24 (66.7%)	0.659^†^
Refractive error (diopters)	0.38 ± 1.64	0.60 ± 1.44	0.551*
Lateral incomitance (n, %)	0 (0.0%)	2 (5.6%)	0.113^†^
Amblyopia (n, %)	6 (14.6%)	5 (15.6%)	0.907^†^
Dissociated vertical deviation (n, %)	1 (2.3%)	2 (5.6%)	0.442^†^
Vertical deviation (n, %)	13 (29.5%)	6 (16.7%)	0.178^†^
Oblique muscle dysfunction (n, %)	7 (15.9%)	9 (25.0%)	0.312^†^
Follow-up duration (months)	49.91 ± 14.83	43.17 ± 26.91	0.160*

Group A = patients who had undergone R&R as primary surgery

Group B = patients who had undergone R&R as reoperation

In the angle of deviation, minus means esodeviation

W4D = Worth-4-dot

Lateral incomitance = change of 5 PD or more in lateral gaze from primary position

Vertical deviation = 5 PD or more hypertropia/hypotropia at primary position

*PD* prism diopters

*Mann-Whitney U test

*Pearson chi-square test

### Angle of deviation

At postoperative 1 day, the mean angle of deviation was −5.18 ± 5.96 PD (esodeviation) in group A and −5.28 ± 3.95 PD in group B, which figures represented no significant difference (*p* = 0.932). However, by postoperative 3 months, the mean angle of deviation in group A had become more exotropic than in group B (group A = 1.64 ± 4.31 PD, group B = −0.11 ± 0.82 PD; *p* = 0.012, Table 3). From postoperative 3 months to final follow-up, a larger amount of exodrift was observed in group A than in group B (*p* < 0.05). At final follow-up, the angle of deviation was 8.89 ± 8.52 PD in group A and 2.50 ± 3.68 PD in group B, which comparison represented a significant difference (*p* = 0.000).

**Table 3:** Postoperative angles of deviation (PD) at distance and near (Distance / Near)

Postoperative day	Group A (*n* = 44)	Group B (*n* = 36)	*P* value^*^
1 day	−5.18 ± 5.96 / -4.42 ± 6.91	−5.28 ± 3.95 / -3.94 ± 4.27	0.932/0.723
1 month	0.16 ± 3.80 / -0.14 ± 3.52	−0.56 ± 1.69 / -0.39 ± 2.01	0.300/0.695
3 months	1.64 ± 4.31 / 1.65 ± 4.64	−0.11 ± 0.82 / -0.44 ± 1.44	0.012/0.011
6 months	2.64 ± 4.94 / 2.65 ± 5.31	0.72 ± 2.35 / 0.61 ± 2.47	0.026/0.037
12 months	4.95 ± 7.10 / 3.88 ± 6.47	1.27 ± 3.16 / 0.68 ± 3.01	0.004/0.023
24 months	7.41 ± 8.36 / 5.74 ± 8.25	2.08 ± 2.39 / 1.48 ± 1.59	0.003/0.004
Final follow-up	8.89 ± 8.52 / 7.74 ± 5.14	2.50 ± 3.68 / 2.02 ± 3.91	0.000/0.000

Group A = patients who had undergone R&R as primary surgery

Group B = patients who had undergone R&R as reoperation

In the angle of deviation, minus means esodeviation

*PD* prism diopters

*Mann-Whitney U test

### Surgical success

Until postoperative 6 months, the surgical success rate was not significantly different (at 3 months, 90.9% in group A and 100% in group B; at 6 months, 79.5% and 91.7%, *p* > 0.05). However, from postoperative 12 months to final follow-up, the surgical success rate in group B was significantly higher than in group A (*p* < 0.05, Table 4). The final success rates were 47.7% and 83.3% in groups A and B, respectively (*p* = 0.001).

**Table 4:** Success rates after surgery in two groups

	Number of patients (success rate, %)		*P* value^*^
Postoperative day	Group A (*n* = 44)	Group B (*n* = 36)	
1 month	41 (93.2%)	36 (100.0%)	0.110
3 months	40 (90.9%)	36 (100.0%)	0.063
6 months	35 (79.5%)	33 (91.7%)	0.131
12 months	32 (72.7%)	33 (91.7%)	0.031
24 months	26 (59.1%)	30 (83.3%)	0.019
Final follow-up	21 (47.7%)	30 (83.3%)	0.001

Surgical success = angle of deviation between 8 PD esodeviation and exodeviation

Group A = patients who had undergone R&R as primary surgery

Group B = patients who had undergone R&R as reoperation

*Pearson chi-square test

### Stereopsis and fusion on Worth-4-dot test

In both groups, postoperative stereoacuity, at each visit, was better than preoperative stereoacuity (*p* < 0.05). At final follow-up, the mean stereoacuity was 55.65 ± 18.54 s in group A and 69.72 ± 65.04 s in group B (*p* = 0.345). The improvement of stereoacuity was observed in 65.9% of patients in group A and 63.9% of patients in group B. Although the deterioration of stereoacuity was found in 4.5% patients in group A and 5.6% of patients in group B, they had a good stereoacuity (≤ 100 s of arc) at pre- and postoperative visit. During early postoperative period (< 1 month), there were 10 patients with postoperative esodeviation of <8 PD who had diplopia and temporary deterioration of stereopsis. However, the diplopia and esodeviation were resolved with recovery of stereopsis after postoperative 1 month. Fusion on Worth-4-dot test, at final follow-up, was observed in 76.3% of patients in group A and 78.1% of patients in group B, which difference was not significant (*p* = 0.857).

### Dose-effect ratio of R&R

**Fig. 1:**
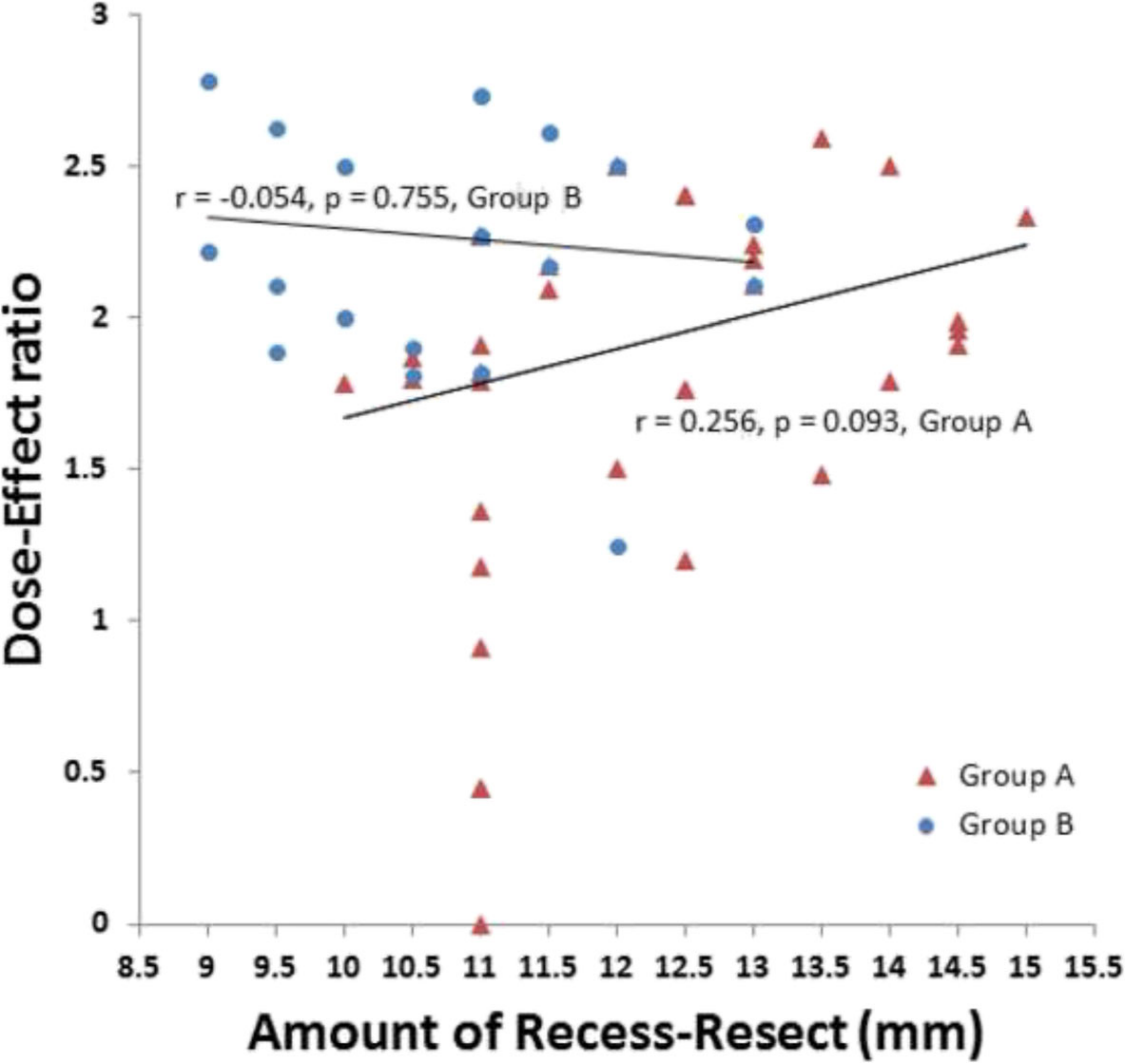
Pearson’s correlation analysis: correlation between amount of recess-resect (mm) and dose-effect ratio (PD/mm) in groups A and B

The ratio of the corrected angles of deviation to the sum of the amount of recession of LR and of resection of MR (dose-effect ratio of R&R) at postoperative 6 months was 1.89 ± 0.58 PD/mm in group A and 2.26 ± 0.32 PD/mm in group B, which difference was significant (*p* = 0.001, Table 5). According to Pearson’s correlation analysis, there was a weak positive correlation between the dose-effect ratio and the amount of recess-resect in group A (*r* = 0.256), but it was not statistically significant (*p* = 0.093, Fig. 1). In group B, there was no correlation between the dose-effect ratio and the amount of recess-resect (*r* = −0.054, *p* = 0.755, Fig. 1).

**Table 5:** Dose-effect ratio at postoperative 6 months according to amount of R&R

Amount of recess-resect	Mean dose-effect ratio (PD/mm)		*P* value
(mm)	Group A (*n* = 44)	Group B (*n* = 36)	
9.0	-	2.50 ± 0.32	
9.5	-	2.21 ± 0.38	
10.0	1.78 ± 0.00	2.25 ± 0.28	
10.5	1.83 ± 0.04	1.85 ± 0.06	
11.0	1.69 ± 0.74	2.27 ± 0.25	
11.5	2.13 ± 0.05	2.46 ± 0.25	
12.0	2.16 ± 0.57	2.08 ± 0.72	
12.5	1.98 ± 0.49	-	
13.0	2.18 ± 0.06	2.24 ± 0.11	
13.5	2.03 ± 0.78	-	
14.0	2.14 ± 0.50	-	
14.5	1.95 ± 0.04	-	
15.0	2.33 ± 0.00	-	
Mean	1.89 ± 0.58	2.26 ± 0.32	0.001^*^

Dose-effect ratio = corrected angle of deviation / amount of recess-resect

Amount of recess-resect = sum of lengths of recession of LR and of resection of MR

Group A = patients who had undergone R&R as primary surgery

Group B = patients who had undergone R&R as reoperation

*Mann-Whitney U test

In the primary surgery group, the mean dose-effect ratio for the 29 patients with fusion was 1.89 ± 0.54 PD/mm, and for the 15 patients with no fusion, 1.89 ± 0.67 PD/mm (*p* = 0.593, Mann-Whitney U test). In the reoperation group, the mean dose-effect ratio for the 24 patients with fusion was 2.27 ± 0.31 PD/mm, and for the 12 patients with no fusion, 2.25 ± 0.35 PD/mm (*p* = 0.671, Mann-Whitney U test). The dose-effect ratio was not found to be significantly associated with the presence/absence of fusion.

## Discussion

In determining the surgical treatment for intermittent exotropia, factors such as age, state of fusional control, and amount of exodeviation are considered [19]. The goals of surgical correction in patients with exotropia are to create a satisfactory ocular alignment and to maintain binocular function. Some patients experience recurrence of exotropia and thus require an additional operation [9–13]. When we encounter patients who are to undergo reoperation for recurrent exotropia in clinical settings, they or their parents usually are concerned about the prognosis and the accuracy of the surgical dosage for avoidance of complications such as over- or undercorrection. These questions prompted us to compare the postoperative outcomes of the same surgical procedure (R&R) as primary surgery and reoperation for intermittent exotropia.

The reported surgical results of R&R as reoperation have been various. Hahm et al. reported that 71% of 58 patients in the reoperation group maintained surgical success without recurrence [15]. In a much more recent study, Kim and Kim, employing survival analysis, showed that the cumulative success rate for recurrent intermittent exotropia after reoperation was 66.4% at final follow-up and decreased over time [16]. In the present study, the success rate, relative to Hahm et al. and Kim and Kim, was higher: 83.3% of 36 patients for R&R as reoperation at final follow-up (mean postoperative follow-up: 43.17 ± 26.91 months) [15, 16]. Further, the degree of exodrift and the recurrence rate were both lower after R&R as reoperation than after R&R as primary surgery, which findings were consistent with Kim and Kim’s study [16].

To the best of our knowledge, there has as yet been no investigation conducted to compare the dose-effect ratio (PD/mm) of R&R between primary surgery and reoperation. So, we aimed to clarify whether there is any difference in dose-effect ratio between R&R as primary surgery and R&R as reoperation. The mean dose-effect ratio of R&R was 1.89 ± 0.58 PD/mm and 2.26 ± 0.32 PD/mm as primary surgery and reoperation, respectively: a significant difference (*p* = 0.001). When we performed R&R with the same surgical dose, the degree of correction was greater in reoperation. Pearson’s correlation analysis showed no significant association between the dose-effect ratio and the amounts of LR recession and MR resection in either group (*p* = 0.093 in group A, 0.755 in group B). The difference of dose-effect ratio between primary surgery and reoperation would result from the horizontal muscle tonus in the contralateral eye. The muscle tone of the contralateral eye in the primary surgery group was not affected. However, the muscle tone of the contralateral eye in the reoperation group had already been affected by the previous operation. Resection of contralateral MR might act as a mechanical force, and recession of contralateral LR would change the arc of contact, which in turn would induce a low postoperative exodrift and a large dose-effect ratio of R&R in a reoperation group [17, 20]. According to Kim et al.’s report, the exodrift changes after a second ULR muscle recession were smaller than those after a first ULR muscle recession in small-angle (< 25 PD) exotropia patients [21]. They explained that the stability of ULR recession as a second surgery is affected by several factors such as age at the time of surgery and the presence of latent exodeviation [21]. In the same manner, our study results might have been affected by these factors. The first factor is age at the time of surgery. In the previous study, lower recurrence rates in older intermittent exotropia patients were observed after unilateral R&R [22]. In our study, the mean age at the time of surgery was 71.70 ± 30.59 and 107.31 ± 40.53 months in groups A and B, respectively. Such difference would be likely to affect surgical outcomes. The second factor is the manifestation of latent exodeviation. At primary surgery, latent exodeviation could remain dormant; however, it appeared after primary surgery. So, we were able to measure the angle of deviation accurately before reoperation.

There are some limitations to our study. First, it was a retrospective study, for which the surgeon determined the surgical modality and time; thus, selection bias might have occurred. Second, there was a difference of preoperative angle of deviation between the two groups. Specifically, the preoperative angle of deviation in the patients with R&R as primary surgery was larger than in those with R&R as reoperation. The reasons were as follows: (1) the patients with R&R as a reoperation had already undergone the primary surgery, which induced a relatively small angle of deviation, and (2) early detection of exotropia recurrence in the patients with R&R as a reoperation could be made, due to their steady follow-up after the primary surgery. A discrepancy of preoperative angle of deviation would be likely to influence the postoperative angle of deviation and surgical success. We believe therefore that an additional, prospective study should be conducted to confirm our present results. In any case, this study would remain a useful comparative case series, given the distinct difference in dose-effect ratio between the two groups. As for the third limitation of this study, the age of patients at R&R as reoperation was older than that of patients at R&R as primary surgery. In some degree, this difference would act as a bias affecting surgical outcomes such as postoperative angle of deviation and surgical success. The fourth and final limitation is the fact that this study enrolled only patients who had undergone R&R as the primary surgery and the same procedure in the other eye as reoperation. Consequently, we were unable to demonstrate whether the R&R procedure is superior, as reoperation, to alternative surgical procedures. In this light, the aforementioned additional prospective study will be suitable for comparison of the surgical outcomes between the R&R procedure and the other surgical methods.

## Conclusions

In summary, unilateral R&R as reoperation showed a better surgical success rate for treatment of recurrent exotropia (83.3%) than R&R as primary surgery (47.7%). The mean effect per millimeter (the dose-effect ratio, PD/mm) of R&R as reoperation for achievement of ocular alignment was 2.26 ± 0.32 PD/mm, which was better than that of primary surgery (1.89 ± 0.58 PD/mm). Therefore, these results could serve as useful guidelines in the planning of surgical correction for primary and recurrent exotropia.

## Abbreviations

DVD: Dissociated vertical deviation
LR: Lateral rectus
MR: Medial rectus
R&R: Lateral rectus recession and medial rectus resection
